# The effect of educational intervention based on social support theory on the perceived stress caused by the covid-19 pandemic in patients with diabetes in hormozgan (2020–2021)

**DOI:** 10.1186/s12889-024-18180-y

**Published:** 2024-03-04

**Authors:** Reihaneh Taheri kondar, Laleh Hassani, Amin Ghanbarnejad

**Affiliations:** 1 MSc of Health Education and Promotion HUMS, Hormozgan University of Medical Sciences (HUMS), BandarAbbas, Iran; 2 Mother and Child Welfare Research Center, Hormozgan University of Medical Sciences, BandarAbbas, Iran; 3 Department of community medicine, research institute for health, Hormozgan University of medical sciences, BandarAbbas, Iran

**Keywords:** Covid-19, Diabetes, Educational intervention, Perceived stress, Social support

## Abstract

**Background:**

People with diabetes are more at risk of covid-19. Perceived social support plays an important role in maintaining people’s health and reducing the negative effects of stress caused by the environment and society. The present study was designed and implemented with the purpose of determining the effect of educational intervention based on social support theory in reducing stress caused by the covid-19 pandemic in people with diabetes.

**Methods:**

The current investigation was an interventional and semi-experimental study conducted on 212 patients diagnosed with type 2 diabetes. Eligible participants were diabetic individuals capable of utilizing virtual platforms and not afflicted with COVID-19. Exclusion criteria encompassed unwillingness to continue study participation, absence from multiple training sessions, and development of a specific illness during the study period. Random allocation placed patients into either the control or intervention group. The intervention group received educational materials via WhatsApp, while the control group did not receive any intervention. The researcher administered a questionnaire to collect demographic information and assess perceived social support among the patients. Data analysis involved the use of chi-square tests, independent and paired t-tests, as well as ANCOVA.

**Results:**

This study revealed that the mean age of patients in the control and intervention groups was 46.35 ± 14.15 and 51.72 ± 11.57, respectively. Most of the diabetic patients in both groups were female, married, had a diploma, were housekeepers, and had an income between 2 and 5 million Tomans. According to the results obtained in all subscales of social support theory as well as the perceived stress score due to the corona pandemic after the educational intervention, a statistically significant difference was observed between the two groups (*P* < 0.05), so that the score of all subscales of social support theory in the intervention group was higher than the control group. But the perceived stress score caused by Corona in the intervention group was significantly lower than the control group.

**Conclusion:**

The results of this study illustrate the noteworthy influence of social support training in lessening perceived stress among patients with diabetes during the COVID-19 pandemic. Consequently, healthcare providers are encouraged to integrate social support education programs into comprehensive care initiatives for diabetic patients, particularly during periods of heightened stress like the current coronavirus pandemic.

## Introduction

Diabetes is a rapidly growing disorder that is prevalent worldwide [1]. The aging global population and the shift towards sedentary lifestyles have contributed to a rapid increase in the number of diabetes patients [2]. As a prevalent chronic disease, it has garnered significant attention from researchers [3]. Additionally, the spread of the coronavirus pandemic has had detrimental effects on individuals with diabetes [4]. It quickly became evident that people with diabetes were disproportionately affected by the virus, facing an elevated risk of hospitalization and mortality [5]. Among patients infected with the coronavirus, diabetes ranked as the second most common comorbidity after cardio-metabolic diseases [6]. During the pandemic, individuals with diabetes experienced more severe illness when infected and required specialized care [7]. Therefore, the stress of the Covid-19 pandemic can impose a greater psychological burden on individuals with underlying conditions such as diabetes due to their physical health status [8]. Given that mental health issues such as anxiety and depression are more prevalent in diabetic patients than in the general population [9, 10], the additional stress brought on by the Covid-19 pandemic and the associated restrictions presents a unique challenge for these individuals. This added stress can exacerbate their mental health disorders [11]. The co-occurrence of diabetes and mental health issues, as opposed to having diabetes alone, can heighten the risk of blood sugar irregularities and the development of diabetes-related complications such as retinopathy, neuropathy, and nephropathy, ultimately leading to a diminished quality of life for these individuals [8, 12]. In one of the studies it was found that more than half of people with diabetes experienced moderate to severe stress during the Covid-19 pandemic, and their main concerns were about blood sugar control and the availability of appropriate care [13]. Lack of support from diabetic care teams, reduced access to health care, and reduced social support lead to increased stress and anxiety of diabetics at risk of contracting Covid-19 [14].

Non-pharmacological interventions, such as patient education, have the potential to alleviate neuropathy symptoms and reduce stress to some extent. There are various methods used to educate patients, but one comprehensive approach is the support training package, which considers the physical and mental dimensions of the patients. Supportive educational programs are essentially a process through which individuals and patients learn behaviors that promote, maintain, and enhance health [15]. Therefore, social support involves the interaction between the provider and the recipient of support [16]. Social support encompasses the care, love, respect, consolation, and assistance that individuals or groups offer to others. This support can come from a variety of sources, including spouses, partners, family members, relatives, friends, colleagues, medical professionals, or social organizations [17]. Essentially, social support encompasses emotional, instrumental, informational, and evaluative forms of assistance. Emotional support focuses on fostering empathetic connections within one’s social network, while informational support involves offering advice, opinions, and relevant information to aid individuals in addressing challenges. Instrumental support is associated with practical assistance and activities [18].. Sharifi Rad et al. conducted a review study on the significance of social support in self-care among individuals with diabetes. The findings indicated that the perceived level of social support among diabetic patients is suboptimal [19]. Additionally, Gillibrand’s study also demonstrated that social support among diabetic patients is not at an optimal level [20]. The study by Gu et al. (2017) emphasizes that social support should be considered a key component in interventions designed to enhance the management of patients with type 2 diabetes mellitus (T2DM) [21].

The Covid-19 pandemic has caused stress and mental conflict for everyone, particularly those at high risk, such as individuals with underlying health conditions like diabetes. This heightened stress can worsen their existing conditions and lead to other health issues. Therefore, it’s crucial for society to raise awareness about the importance of social support for diabetic patients and educate both patients and their families on stress management, as this can significantly improve the quality of life for these individuals. Considering the findings from studies on the relationship between social support and stress reduction, as well as the correlation between perceived stress in individuals with diabetes and the susceptibility to contracting Covid-19, and recognizing the absence of studies addressing these topics in the context of the Covid-19 pandemic, this study was devised and executed with the objective of assessing the impact of an educational intervention based on social support theory on the perceived stress caused by the covid-19 pandemic in patients with diabetes.

## Methods

### Study design and setting

The present study was a semi-experimental intervention type which was performed in 2020–2021. The study population consisted of diabetic patients under the coverage of Bandar Abbas Diabetes Clinic and Comprehensive Health Service Centers of Rodan city, who had family files and were registered in the Sib system.

### Sample size and sampling

The eligible participants were chosen based on the study’s inclusion criteria and using an available sampling method. Inclusion criteria comprised being diabetic, not having Covid-19, having the capability to use virtual platforms, and possessing literacy skills. Exclusion criteria encompassed unwillingness to continue participation, missing more than one training session, and the onset of a specific disease during the study period. The required sample volume was calculated according to the below formula: (Based on previous studies [22] and considering the standard deviation of 11.1, considering the error of 5%, the test power is 80% and the effect size is d = 4.5)

$$ n=\frac{2{\left({Z}_{1-\raisebox{1ex}{$\alpha $}\!\left/ \!\raisebox{-1ex}{$2 $}\right.}+ {Z}_{1-\beta }\right)}^{2}{s}_{p}^{2}}{{d}^{2}}=96$$

To mitigate potential dropouts, 10% was added to the initial sample size. Consequently, the final sample size for each of the two intervention and control groups was calculated to be 106 individuals, resulting in a total estimated study sample size of 212 people. Then, the patients referred to Bandar Abbas diabetes clinic were selected as the intervention group and the patients from two other centers in Rodan city were selected as the control group. By selecting patients from different centers, the researchers were able to more effectively control for potential confounding factors, such as age, gender, and socioeconomic status, which may be specific to a particular clinic or location. This approach allowed for a more accurate assessment of the true effect of the intervention on patient outcomes.

### Measurement

The data collection tool comprised three parts: demographic information, social support, and perceived stress as per Cohen’s scale. The second and third parts of the tool were specifically related to Covid-19.

The first part of the questionnaire includes 19 demographic questions such as age, gender, marital status, level of education, history of diabetes, etc.

The second part of the researcher-designed questionnaire was structured around four subcategories of social support, aiming to assess the emotional, informational, instrumental, and appraisal support received by individuals in relation to diabetes during the Covid-19 pandemic. The emotional support section comprised 9 questions rated on a Likert scale (ranging from “never” to “always” with a total score range of 9–45). The informational support section included 7 questions rated on a Likert scale (ranging from “never” to “always” with a total score range of 7–35). The instrumental support section consisted of 9 questions rated on a Likert scale (ranging from “never” to “always” with a total score range of 9–45), and the appraisal support section encompassed 7 questions rated on a Likert scale (ranging from “never” to “always” with a total score range of 7–35). The validity of the tool was established using the expert panel method, involving the distribution of a questionnaire to experts in the fields of health education and health psychology. Following the receipt of responses from the panel participants, the Content Validity Ratio (CVR) and Content Validity Index (CVI) were calculated (CVI = 0.935, CVR = 0.892). Reliability was determined using the test-retest method, where the questionnaire was administered to 20 individuals at a 10-day interval. A strong and statistically significant correlation was observed for the social support questionnaire (*r* = 0.967, *p* < 0.001).

The third part was related to measuring the perceived stress of people with diabetes during the covid-19 pandemic, and the 10-question Cohen Perceived Stress Questionnaire was used for the covid-19 pandemic [23]. Perceived stress tool scale which was 5 Likert (completely agree to completely disagree) had a range of 0 to 40 and four questions were scored inversely and getting a higher score was a sign of high level of perceived stress of the participants in the study, and the score of equal and more than 25 was considered as high stress and the lower values were considered as low stress.

Cohen et al. (1983, cited by Mahmoudpour et al., 2017) calculated its correlation coefficient with semiotic measures between 0.52 and 0.76 to calculate the validity of this scale [24]. In the research of Behrouzi, Shahni Yeylagh & Pourseyed (2011), Cronbach’s alpha coefficient and Tasneef were used to calculate the reliability of perceived stress, and the values of 0.73 and 0.74 were obtained, respectively [25]. Abolghasemi and Narimani (2004) translated and standardized this questionnaire in Iran, and its validity and reliability have been assessed as appropriate [26]. In the research of Lotfi et al. (2022), reliability was estimated to be 0.76 using Cronbach’s alpha method [27]. To determine the reliability of the tool in this study, the questionnaire was distributed and completed among 20 people in the sample randomly and with an interval of 10 days in two occasions. In the results of the test-retest, it was found that there was no statistically significant difference in the scores of the questionnaire questions in the two times (*p* < 0.05). The internal reliability of the questionnaire from Cronbach’s alpha is α = 0.87.

### Data collection

To conduct the research, the researcher obtained the code of ethics from the Ethics Committee of Hormozgan University of Medical Sciences and secured permission from relevant officials. Due to the challenges posed by the COVID-19 pandemic and the necessity of adhering to social distancing measures, the researcher employed a simple random method to select eligible individuals from the electronic records in the Sib system, in accordance with predetermined criteria. In order to control confounding factors and given the difficulties associated with in-person interactions during the pandemic, the decision was made to select study participants from separate centers. Consequently, patients in the intervention group were chosen from the diabetes clinic at Hormoz Center in Bandar Abbas city, while patients in the control group were selected from two comprehensive urban health service centers in Rodan city. The choice of Rodan Center was motivated by the geographical distance, ensuring minimal contact between the test and control groups. Subsequently, the researcher extracted patient information, including contact details and demographic data, from their files and obtained verbal informed consent from the selected individuals to participate in the study via telephone calls. The patients were then asked to respond to the study questionnaires orally over the phone, constituting the pre-test stage. The intervention was implemented using the WhatsApp Business application, chosen for its widespread use, accessibility, and user-friendly features. A WhatsApp group was established, comprising 106 diabetic patients in the intervention group, through which the study’s objectives, methodology, and progress were communicated during multiple sessions. At the outset, the group received the project’s objectives and essential explanations in the form of an audio file. The educational program and objectives were then elucidated and presented to the participants through visual aids. The daily program followed a consistent schedule, commencing each morning with a motivational message, accompanied by a picture or video, and a greeting. This routine aimed to impact the emotional dimension, with the inclusion of scenic images and clips of Iranian cities, reflecting the prevailing societal conditions amidst the pandemic and travel restrictions, as well as people’s interest in such content. Subsequently, the primary content of the day, comprising photos and videos, was shared in the group, along with a daily question and its answer. On Fridays and Saturdays, clips produced by the Ministry of Interior for COVID-19 were featured, while weekends and holidays saw the inclusion of posters encouraging individuals to stay at home, alongside educational posts. Additionally, instructional materials were posted in the evenings to complement ongoing topics. The topics, detailed in Table 1, were each taught over one-week periods, with the training program spanning three months. To ensure participants’ engagement, weekly online sessions were conducted, allowing for discussions and addressing any queries. Participants who were unable to attend were contacted individually to gather their feedback. In addition to group training, each individual received four 15-minute phone sessions. Subsequently, a post-test was conducted, and the questionnaires were completed once more. The control group did not receive any specific intervention and only had access to publicly available information and support from media, society, and healthcare providers. A post-test was also conducted for this group after three months. The collected results were then analyzed.

**Table 1:** Chapters of content posted during the intervention

	Title
1	Understanding COVID-19 and Its Symptoms
2	Preventing COVID-19 and Principles of Care in Various Settings
3	The Significance of Care for Individuals with Diabetes
4	Defining and Identifying Stress as a Medical Condition
5	The Impact of Stress on Diabetes
6	Methods for Managing Stress
7	The Significance of Physical Activity During the COVID-19 Pandemic and Its Role in Stress Reduction
8	Creating a Homemade Face Mask
9	Dietary Considerations for Diabetics During the COVID-19 Pandemic
10	Children During the COVID-19 Pandemic
11	University of Medical Sciences News, Announcements, and Essential Information
12	Extracurricular Content Including World Hand Hygiene Day, World Blood Pressure Day, Measles Outbreak, Vaccination Schedule Follow-Up for Various Groups, Thyroid Day, and More
13	Group Activities, Such as Hand Hygiene Contests for Prevention Discussions, Healthy Plate Challenges for Nutrition Education, and Coordinated Picture Posts with Educational and Support Objectives

### Data analysis

The data was analyzed using SPSS Version 22 software. The results were presented as frequency (percentage) and mean ± standard deviation. The Shapiro-Wilk test was employed to assess the normality of the distribution of quantitative variables. Additionally, various inferential statistical methods were utilized for data analysis, such as the Chi-square test, Fisher’s exact test, t-student test, paired-samples t test, and analysis of covariance (ANCOVA). A significance level of *P* < 0.05 was considered statistically significant.

## Results

According to the results, in this research, the average age was 46.35 ± 14.15 years in the control group and 51.72 ± 11.57 years in the intervention group. Most of the participating diabetic patients in both groups were female (57.5% in the control group − 56.6% in the intervention group), married (73.6% in the control group and 85.8% in the control group), and had a diploma (38.7% in the control group and 30.2% in the intervention group), were housekeeper (39.7% of the control group and 39.6% of the intervention group), had income between 2 and 5 million Tomans (43.4% of the control group and 60.4% in the intervention group), with a history of heart disease (25.5% in the control group and 30.2% in the intervention group) and history of first degree relatives were in both groups. The intervention and control groups showed a statistically significant difference in terms of spouse’s occupation, income, number of underlying diseases, and history of close relatives among the mentioned variables (*P* < 0.05) (Table 2).

**Table 2:** Demographic characteristics of study participants

Variable		Control - Rodan number (percentage)	Intervention - Bandar number (percentage)	*P*-value*	Variable		Control - Rodan number (percentage)	Intervention - Bandar number (percentage)	*P*-value*
Sex	Man	(5/42)45	(4/43)46	89/0	Wife’s Job	Single	(16)17	(2/13)14	^*^025/0
	Female	(5/57)61	(6/56)60			Employee	(17)18	(3/12)13	
Marital	Single	(5/8)9	(7/5)6	^*^159/0		housewife	(4/26)28	(33)35	
	Married	(6/73)78	(8/85)91			household income	(8/2)3	(8/2)3	
	A man or a woman without a partner	(7/4)5	(8/2)3			free	(9/18)20	(3/12)13	
	Widow	(2/13)14	(7/5)6			Retired	(8/2)3	(3/12)13	
Education	Elementary	(17)18	(7/21)23	590/0		Farmer	(6/7)8	(7/3)4	
	Tip or cycle	(6/22)24	(5/25)27			Pensioner	(8/3)4	(0)0	
	Diploma	(7/38)41	(2/30)32			manual worker	(7/4)5	(4/10)11	
	College Education	(7/21)23	(6/22)24		Income	Under 2 million tomans	(4/42)45	(8/19)21	002/0
Wife’s education	Single	(16)17	(2/13)14	257/0		Between 2 million and 5 million tomans	(4/43)46	(4/06)64	
	Illiterate	(5/8)9	(8/2)3			Above 5 million tomans	(2/14)15	(8/19)21	
	Elementary	(2/13)14	(2/13)14		History of underlying disease	Yes	(9/50)54	(3/44)47	336/0
	Tip or cycle	(2/14)15	(6/24)26			No	(1/49)52	(7/55)59	
	Diploma	(1/32)34	(33) 35		Name of underlying disease	heart	(5/25)27	(2/30)32	^*^034/0
	College Education	(16)17	(2/13) 14			thyroid	(5/6)7	(8/3)4	
Job	Employee	(8/18)21	(3/11) 12	^*^23/0		Thalassemia	(7/5)6	(0)0	
	Housewife	(7/39)42	(6/39) 42			Asthma	(9/0)1	(9/1)2	
	Household Income	(7/4)5	(7/5) 6			blood fat	(6/6)7	(5/8)9	
	Free	(17)18	(4/10)11			kidney	(8/2)3	(0)0	
	Retired	(5/7)8	(6/24)26			liver disease	(8/2)3	(0)0	
	Farmer	(9/0)1	(9/0)1		History of close people	first degree relatives	(5/58)62	(1/48)51	008/0
	Pensioner	(9/2)3	(9/0)1			second degree relatives	(8/19)21	(3/11)12	
	Manual Worker	(5/7)8	(6/6)7						

* Fisher’s exact test

The results in Table 3 indicated that the average perceived stress in the control group was 17.51 ± 6.97 before the intervention and 23.13 ± 7.89 after the intervention. Based on the results of the statistical test, a statistically significant difference was found between the average score of perceived stress in the control group before and after the intervention, indicating an increase in stress levels after the intervention (*P* < 0.0001). The average perceived stress score in the intervention group was 22.23 ± 2.91 before the intervention and 10.84 ± 4.72 after the intervention. According to the results of the statistical test, there was a statistically significant difference between the average score of perceived stress before and after the intervention in the intervention group, with the amount of stress decreasing after the intervention (*P* < 0.0001). Additionally, the results indicated a significant difference in the average score of perceived stress between the two intervention and control groups, with the stress level in the intervention group being lower than that in the control group after the intervention (*P* < 0.0001).

**Table 3:** Comparing the perceived stress scores related to the COVID-19 pandemic between the control and intervention groups separately before and after the educational intervention

variable\ group	control		intervention		***P*** -Value**
	Before the educational intervention	After the educational intervention	Before the educational intervention	After the educational intervention	
Perceived stress caused by the corona epidemic	97/6 ± 51/17	89/7 ± 13/23	91/2 ± 23/22	72/4 ± 84/10	001/0>
P-Value*	0001/0>		0001/0>		

* Related to paired t results

** Corresponding to independent t results

The results of the present study indicated that in the control group, the average score of the components of emotional and instrumental support significantly decreased after the intervention (*P* < 0.0001). Conversely, in the intervention group, the average score of all social support components after the intervention was significantly higher than before (*P* < 0.0001). Furthermore, the results revealed a statistically significant difference between the two intervention and control groups in terms of all social support components, with the scores of the intervention group being significantly higher than those of the control group (*P* < 0.0001) (Table 4).

**Table 4:** Comparing the components of the social support theory between the control and intervention groups separately before and after the educational intervention

variable\ group	control		intervention		***P*** -Value**
	Before the educational intervention	After the educational intervention	Before the educational intervention	After the educational intervention	
Emotional support	34/5 ± 47/32	17/5 ± 46/31	08/6 ± 05/29	03/4 ± 72/36	001/0>
***P*** -Value*	0001/0>		0001/0>		
Information support	75/3 ± 71/20	19/3 ± 67/20	09/5 ± 54/22	69/2 ± 45/30	001/0>
***P*** **-Value**	873/0		0001/0>		
Instrumental support	99/5 ± 64/34	14/5 ± 06/33	65/6 ± 31/30	58/4 ± 99/34	001/0>
***P*** **-Value**	0001/0>		0001/0>		
Evaluation support	54/4 ± 68/23	28/4 ± 38/23	11/5 ± 39/23	19/3 ± 83/28	001/0>
***P*** **-Value**	157/0		0001/0>		

* Related to paired t results

** Corresponding to independent t results

The results of the covariance analysis (Table 5) revealed that the change in the scores of all social support variables and the perceived stress score due to the corona pandemic in the intervention group was higher than in the control group after controlling for the pre-test score. Before the educational intervention, a score greater than or equal to 25 was considered as high stress, while a score less than 25 indicated low stress. There was no difference in the level of stress caused by the corona pandemic between the participants of the two groups (*p* = 0.853). However, after the educational intervention, in the intervention group, the percentage of people with a low stress level increased from 84% to about 97%, and those with a high stress level decreased from 16 to 2.8%. In contrast, in the control group, after the educational intervention, the percentage of people with a high level of stress increased from 17% to about 48% (*P* < 0.0001). According to McNemar test, a significant difference was observed in terms of changes in stress levels caused by the corona pandemic after the educational intervention compared to before the educational intervention in both control and intervention groups (*P* < 0.0001 and *P* = 0.001, respectively) (Table 6).

**Table 5:** The results of covariance analysis related to the perceived stress caused by the corona epidemic and the components of social support theory

Variable	Sum of squares	Degrees of freedom	Average of squares	Test criteria	***P*** **-Value**
Emotional support	84/574	1	84/574	29/108	0001/0>
Information support	98/492	1	98/492	15/115	0001/0>
Instrumental support	65/299	1	65/299	77/45	0001/0>
Evaluation support	10/437	1	10/437	23/97	0001/0>
Perceived stress caused by the corona epidemic	61/244	1	61/244	95/10	001/0

**Table 6:** Comparing the levels of perceived stress caused by the social corona epidemic between two control and intervention groups separately before and after the educational intervention

**Measurement time**	**Group**	**Stress Level**		***P*** **-value**
		**Low**	**High**	
Before the educational intervention	Control (Rodan)	88 (83)	18 (17)	853/0
	Intervention (Bandar)	89 (84)	17 (16)	
After the educational intervention	Control (Rodan)	55 (9/51)	51 (1/48)	0001/0>
	Intervention (Bandar)	103 (2/97)	3 (8/2)	

## Discussion

The purpose of the present study was to determine the role of educational intervention based on the social support model in reducing stress caused by the covid-19 pandemic in people with diabetes. In the current study, the educational intervention based on the social support model had a significant effect on reducing the perceived stress caused by the pandemic in the intervention group compared to the control group. Researchers believe that people who have less stress are better able to adapt to life events and those who have higher stress are less able to adapt and even some behaviors such as aggression are more among these people [28]. Considering that one of the main problems of patients with diabetes is perceived stress, and in today’s special conditions, where these people are under additional stress due to the disease of Covid-19, helping them to reduce stress and improve their condition are necessary and unavoidable.

In addition to the physical effects, the stress caused by diabetes also has adverse psychological effects that make it difficult to treat and control diabetes. Some researchers consider stress management group training to be effective in reducing negative emotions and increasing the sense of self-efficacy and hope of diabetic patients [29]. Sanjeev et al. (2010) determined that the implementation of stress management reduces blood sugar and depression in women with type 2 diabetes, and these findings remained stable after a one-year follow-up period [30]. Hamid (2011) also demonstrated that stress management training reduces glycosylated hemoglobin and reduces depression, anxiety and stress in type 2 diabetics [31].

Badaghi et al. showed that anxiety and stress symptoms have an inverse relationship with social support [32]. The role of social support in coping with stressful events was investigated in the study of Gonosen et al., and it was observed that the social support of colleagues and family to people under difficult circumstances makes it easier for them to cope with stressful situations [33].

People in different stages of stress caused by illness need a specific type of social support, so that in the crisis stage, people need emotional support, in the stabilization stage, they need informational support, and in the fatigue stage, they need instrumental support [34]. Nabavi et al. indicated the role of social support and its dimensions to be effective on the general health of the elderly with diabetes [35]. Motamedi Shalmazari et al. found that social support had a significant relationship with life satisfaction, general health, and loneliness in diabetic patients [36]. The result of Chan et al. and Lee et al., showed that social support is the most powerful coping force for the successful and easy coping of people involved with chronic diseases and stressful conditions, and enhances the patient’s tolerance [37, 38].

Social support can reduce the adverse effects of chronic disease and help patients to adapt better to their disease [39]. The results showed that there is a significant positive relationship between perceived social support and stress reduction caused by the covid-19 pandemic. This result is consistent with the findings of Chan et al. and Lee et al., in which social support is the most powerful coping force for the successful and easy coping of people involved with chronic diseases and stressful conditions, and enhances the patient’s tolerance [37, 38]. People in different stages of stress caused by illness need a specific type of social support, so that in the crisis stage, people need emotional support, in the stabilization stage, they need informational support, and in the fatigue stage, they need instrumental support [34]. Nabavi et al. indicated the role of social support and its dimensions to be effective on the general health of the elderly with diabetes [35]. Motamedi Shalmazari et al. found that social support had a significant relationship with life satisfaction, general health, and loneliness in diabetic patients [36].

The results of the present study revealed a statistically significant difference between the two intervention and control groups in terms of all social support components, with the scores of the intervention group being significantly higher than those of the control group. According to studies, 20 to 40% of patients with diabetes experience emotional distress [40, 41]. Motamedi Shalmazari et al. reported that emotional support plays a more important role than informational and instrumental support [36]. According to Sitner (2018), preventing stress and distress in diabetics plays an important role in managing the disease and reducing the complications caused by the disease [42]. Also, Downey et al. (2021) demonstrated that emotional support can have a close relationship with monitoring the blood sugar in diabetics [43]. Therefore, it is necessary for health professionals to take effective steps in order to increase emotional support in diabetic people [44].

The results of some studies show that lack of awareness and not having enough informational support causes fear and excitability of patients [45, 46]. Therefore, providing a series of information and training in the enhances the awareness about the field of performing certain skills of how to take care of themselves, disease control and treatment regime, and their anxiety, concern and mortality rate will decrease [47]. Khodapanahi et al. (2010) showed that information can increase the control of patient perceptions by providing ways to manage the disease and deal with the symptoms [47].

In terms of investigating the dimension of instrumental support and informational support, Cheraghi et al. (2015) investigated the role of perceived social support in heart failure patients and observed that women percieve less informational and instrumental support than men. While previous studies show that men and women do not differ in understanding informational and tangible support [48]. In the present study, apart from the issue of gender, it was observed that the educational intervention increases the instrumental support in the subjects under study, which is in line with Cheraghi’s study.

The study’s strengths lie in its clear demonstration of the significant impact of social support training on reducing perceived stress among patients with diabetes during the corona epidemic, making it particularly relevant in the current global health crisis. The findings emphasize the importance of addressing both physical and mental well-being and offer practical implications for healthcare providers, suggesting the integration of social support education programs into comprehensive care programs for diabetic patients, especially during times of high stress such as the coronavirus pandemic. Overall, the study suggests that social support interventions have the potential to enhance resilience and improve the quality of life for individuals facing similar health challenges.

The study had several limitations. Firstly, it only included a specific group of patients with diabetes, so generalizing the results to the entire population of diabetic patients should be done cautiously. Additionally, the challenges posed by the spread of Covid-19 and social distancing made sampling difficult, and non-attendance made it harder to gain the trust of patients. Furthermore, the method of filling out the questionnaires through interviews may have led to inaccuracies in the responses.

## Conclusion

In conclusion, the findings of this study demonstrate the significant impact of social support training on reducing perceived stress among patients with diabetes during the corona epidemic. The results underscore the pivotal role of social support as a powerful resource for individuals grappling with chronic diseases and stressful circumstances. By facilitating problem tolerance, mediating the effects of stress on physical and mental well-being, and enhancing cognitive strength, social support not only mitigates tension but also contributes to improved health outcomes and overall quality of life. These insights emphasize the importance of integrating social support interventions into the care and management of individuals facing similar health challenges, offering a promising avenue for enhancing their well-being and resilience in the face of adversity. Health care providers are therefore advised to consider social support education programs in comprehensive care programs for diabetic patients, especially in times of high stress such as the coronavirus pandemic. Hence, healthcare systems and communities can better support people with chronic diseases such as diabetes in managing stress and improving their overall well-being.

## Data Availability

All data generated or analysed during this study are included in this published article.
